# Demonstration of enhanced long-range cosmic time synchronization for wireless and secure dissemination of standard time

**DOI:** 10.1038/s41598-023-49493-4

**Published:** 2024-01-04

**Authors:** Hiroyuki K. M. Tanaka

**Affiliations:** 1 University of Tokyo, Tokyo, Japan; 2 International Virtual Muography Institute (VMI), Global, Tokyo, Japan

**Keywords:** Geophysics, Experimental particle physics, Civil engineering, Electrical and electronic engineering, Environmental impact, Energy and society, Environmental economics, Socioeconomic scenarios, Sustainability

## Abstract

Accurate traceability of time is prerequisite to the proper functioning of many necessary aspects of our modern life including making financial transactions, managing automated technology and navigating the transport of goods and human beings. One of the most reliable international time references is the Coordinated Universal Time (UTC) that can be distributed wirelessly in principle. However, this wireless option is currently limited to GPS and other global navigation satellite systems. GPS signals are weak and easily affected by environmental conditions. Moreover, since GPS signals are unencrypted, the possibility of a signal spoofing attack remains a continuous threat. Prior works showed the potential of the alternative wireless time synchronization technique called Cosmic Time Synchronization (CTS), in which, clocks are located 50 m apart were wirelessly synchronized with a sub-microsecond level accuracy, and its operation time was limited to 20 min. However, for the actual implementation of CTS to real-life situations, these distance and stability values are not sufficient. In this study, we constructed a dedicated CTS facility and conducted a long-haul (180 m) CTS demonstration. As a result, it was verified that this long-range CTS is capable of maintaining stable sub-microsecond time synchronization for 3 days with a granularity of 148.8 ns (SD) and an offset of 22.97 ns. Since the current version of CTS can now operate over an area that has been enlarged by more than one order of magnitude, it is possible to utilize for more diverse applications, and the application to a banking synchronization system is proposed. As a case study, it is shown that CTS now has the capability to offer wireless time synchronization service to large clusters of financial firms in large cities. With its accurate time dissemination (the metrological traceability to UTC), its reasonable cost, and its hack-proof, stable design, this latest CTS model has the capacity to improve the accuracy of timing for a wide variety of sectors.

## Introduction

Accurate time has been recognized as an irreplaceable component for maintaining and operating modern infrastructure, including the electrical power sector^1^, the financial sector^2^, and wireless sensor networks in industries^3^ and hospitals^4^. In many of these sectors, traceability to international standards is required by legislation or regulation. For example, in 2017, the Commission Delegated Regulation of the EU (MiFID II) required time stamping accuracy to be (with respect to Coordinated Universal Time (UTC)) within one millisecond and 100 microseconds (with 1 microsecond granularity) respectively for standard electronic trades and high-frequency trades^5,6^.

Global Positioning System (GPS) is widely used (in conjunction with Galileo, GLONASS and NaVIC) to confirm compliance of atomic-clock-level timing with UTC standards^7,8^. However, GPS satellite signals cannot be used in indoor/underground/underwater environments. Also, GPS signals are affected by environmental conditions such as urban canyon effects and solar storms. An even more important issue is that GPS signals are weak and unencrypted, so jamming/spoofing of the signals remains a continuous threat^9,10^. The most common malicious attack method, directed at financial services in particular, is GPS time source “spoofing”^11^.

Another technique uses dedicated transmission links in communication satellites. This technique is called two-way satellite time and frequency transfer (TWSTFT)^12,13^. It is possible to get nanosecond-level accuracy with TWSTFT which is also traceable to UTC, however, it requires costly dedicated leasing of satellite communication links. Moreover, in either case (with both GPS and TWSTFT), they require wiring connections between the antenna on the roof and throughout the inside structure of each building to enable this time service throughout the building. Constructing and maintaining these infrastructure modifications is costly and time consuming. Therefore, a method which could wirelessly transfer (UTC compatible) accurate timing to clients located inside buildings would drastically improve the availability of this system^14^.

In the UK, a new service was proposed by the National Physics Laboratory to bypass these satellite-based techniques by sending UTC time directly from the atomic clock at Teddington to the London Financial City data centers via a fiber optical link^15^. This service transfers IEEE-1588 Precision Time Protocol (PTP) signals from commercially available PTP grand masters to be delivered through dark fibers (unused optical fiber, available for fiber-optic communication) or dedicated fibers for enhanced communication speed and cyber security. Another example includes the INRIM’s fiber link that was deployed between the financial district in Milan and National Metrology Institute of Italy (INRIM) in Turin for UTC time dissemination^16^. This methodology offers the most traceable, accurate and secured option. However, these dedicated lines are not always available, and if such infrastructures have to be privately installed or replaced for each sector node, the cost will be extraordinary. If UTC could be wirelessly transferred to clients located near these dark fiber/dedicated fiber stations, availability of this system would be drastically improved.

In 2022, Cosmic Time Synchronization (CTS) was proposed by Tanaka^17^ and its first laboratory scale, short haul demonstration was attempted^18^. CTS utilizes multiple electromagnetic particles (EAS particles) included in extended air shower (EAS) events which almost simultaneously and continuously arrive on the Earth’s surface worldwide. Since variations of arrival times of EAS particles are below one microsecond, as long as they land within the same shower disk, the local clocks associated with the detectors (CTS sensors) can be synchronized wirelessly with sub-microsecond accuracy. High-energy interactions between the primary cosmic rays and atmospheric nuclei generate cascades of secondary particles that include electromagnetic and hadronic components. These cascade showers are called the extended air shower (EAS) and they spread out over large areas. The lateral distribution depends on the mass of primary cosmic rays. Heavier primary particles such as Pb generate a flatter lateral distribution but in practical terms, as an approximation, all of the EASs are generated by protons and alpha particles since majority of the primary cosmic rays consist of protons and alpha particles (74% are protons and 18% are alpha particles.) Muons, one of the shower particles, have a decay constant of 2.2 microseconds, but due to their strong relativistic effect and their stronger penetration capability, many of them arrive the ground surface. On the other hand, electrons/positrons with longer path lengths tend to be absorbed more in the atmosphere, so usually cannot reach the ground. As a result, the lateral distribution of electrons/positrons are narrower than that of muons. Tanaka et al.^18^ conducted Monte-Carlo-based (MC-based) EAS simulations initiated by the primary cosmic rays with an energy range below 1 PeV to evaluate the rate of the dual and triple coincidence of the EAS particles as a function of the distance between the detectors. The electron/positron coincidence rate was one order of magnitude higher than muons, but as was expected, the coincidence rate of electrons/positrons was more quickly reduced as a function of the distance between the detectors than the coincidence rate of muons. Furthermore, it was found that the coincidence rate of gamma rays was one order of magnitude higher than electrons/positrons. In the current work, the results acquired from the experimental studies are compared with the numerical results calculated in the prior work.

Because the first iteration had to be done within a laboratory building, the nodal distance between clocks was limited to 50 m in this experiment. If the CTS range is limited to 50 m, the CTS application range would be restricted. In this work, a dedicated CTS facility was constructed by using three independent buildings built at different locations. As a result, the nodal distance could be extended to almost four times (180 m) the CTS range of the first experiment. With the extension done in the current work, the area of CTS coverage was increased by more than one order of magnitude in comparison to the previous POC results. Moreover, in its first iteration, the duration of the measurement was limited to 20 min and thus, CTS stability could be confirmed only for a short period. Obviously, a longer time period (> days) stability is necessary to confirm if real-world and practical applications are feasible. In this paper, it will be shown that CTS wireless time synchronization will fulfill the requirements of MiFID II even at a distance of 180 m. It will also be shown that this improved CTS range will be able to cover and offer wireless clock synchronization service to a large cluster of financial sectors associated with each of the stock exchange districts in large cities like New York, London, and Tokyo. By operating CTS in tandem with at least one dedicated fiber optic line, a resource already part of the infrastructure in most international stock exchanges, UTC can be disseminated (entirely or partly) wirelessly throughout these financial clusters. Additionally, it will be shown that without requiring additional infrastructure, CTS has added benefits: it provides enhanced security against malicious third-party attacks at low costs.

## Results

### Experimental setup

The current CTS server (CS) system consists of a GPS-DO (Protempis Thunderbolt™ PTP GM200), a high voltage supply (HV), a scintillation detector that consists of a photomultiplier tube (PMT) connected with a plastic scintillator sheet via an acrylic light guide, and associated electronics. The CTS client (CC) consists of the same devices except that it incorporates an independent local oven-controlled crystal oscillator (OCXO) instead of GPS-DO. The PMT output is discriminated and fed into the time to digital converter (TDC). The TDC measures the time interval (*t*_i_ where *i* is the event number) between the moment of PMT output and the moment when the closest edge of the 10-MHz TTL pulses outputted from the local OCXO. The time range of the current TDC (ScioSense GPX2) is sufficiently long (> 1 microsecond) to measure the period of these 10-MHz pulses. By counting the number of 10-MHz pulses (*N*_10_), and adding *t*_i_ to *N*_10_ × 100 ns, the muon’s time of arrival at the CS/CC can be acquired^17,18^. As is described later, *N*_10_ is reset every time CS/CC detects the event. The CC clock strategy used for correction of the client time will also be explained later. In the current experiment, the size of the scintillator is relatively large (1 × 1 m^2^) and therefore, it generates an uncertainty value of 6 ns at its maximum; this value is equivalent to the time a photon travels between one corner of the scintillator (directly outside of the PMT photocathode) and the other diagonal corner. More specifically, in this experiment this distance measures 1.4 m, and the photons travel through material with a refractive index of 1.5. However, this uncertainty is much smaller than the targeted timing accuracy (sub-microsecond) and thus, it is neglected. The jitters coming from other sources (e.g., scintillation light rise time, PMT, and discriminators) are all within 1 ns^19^.

The current CTS clock correction procedure is summarized below:

(1-1) The TDC associated with the CS counts the number of 10-MHz pulses outputted from the GPS-DO.
(1-2) The TDC associated with the CC counts the number of 10-MHz pulses outputted from the OCXO.
(1-3) When the detector associated with either the CS or the CC detects the particle event, the discriminated pulse is transferred to the TDC stop input. The TDC measures the time difference (*t*_i_) between the pulse edge generated by this particle event and that of the 10-MHz pulse registered right before this particle event.
(1-4) The CS and the CC add the time difference derived by the (1-3) process to the total count number of 10 MHz pulses (*N*_10_) measured in the processes (1-1) and (1-2), and they record them as their event detection times.
(1-5) This timing information (*t*_i_ + *N*_10_ × 100 ns) is transferred from the CS to the CC via Wi-Fi, and verified by (1-6).
(1-6) If the time difference between the event at the CS and the event at the CC (|[*t*_i CS_ + *N*_10 CS_ × 100 ns]−[*t*_i CC_ + *N*_10 CC_ × 100 ns]|)is within *T*_w_ = 2 microseconds, these events are considered as being events in the same EAS, and this time difference is subtracted from (or added to, depending on whether this time difference is positive or negative) the CC to adjust the time at the CS.

Since highly versatile and commercially available personal computers were used for data transfer from CS to CC, the time required for this entire process from (1-1) to (1-6) was up to 1 s. However, since OCXO drifts only ~ 1 ns in 1 s, this processing time is negligible. On the other hand, since the average time interval of EAS initiated by high energy primaries (> > TeV) (which can generate relatively large shower disks) is much longer than this time value^20^, the granularity and the time stamping accuracy are both largely depending on the frequency of the EAS that can cover a large area; hence the CTS accuracy depends on the nodal distance between the CS and the CC. Also, since relative variations in the arrival time between the detectors are measured, the temporal delays associated with the longitudinal distribution of the EAS particles is measured as fluctuations in CTS time.

The quality of the dissemination of GPS time with CTS depends on the quality of the time synchronization between the 1-pulse-per-second (PPS) reference signal and the 10-MHz reference signal outputs and the 1-Hz serial data output which is synchronized with the National Marine Electronics Association (NMEA) data output. Although GPS time is not exactly the same as UTC, the reference time stamping procedure with a GPS-DO can be directly applied to UTC, and is summarized here. With the GPS-DO, the reference time is outputted in an NMEA format via a serial port. These NMEA data consist of latitude, longitude, altitude, date, and time. 1-PPS/10-MHz pulses can be labeled by the GPS time by taking a coincidence between the 1-Hz NMEA output. Therefore, with CTS, GPS time is registered to the CS in the following way:

(3-1) Search for a coincidence event between the 1-Hz serial pulse and the 1-PPS/10-MHz TTL pulses.
(3-2) Register the NMEA data to the specific address of 1-PPS/10-MHz TTL pulses. Here every 1-PPS pulse and every one out of 10^7^ 10-MHz pulses are labeled with the GPS time.
(3-3) Add the GPS time derived by the process (3-2) to the time interval derived by the process (1-3) (*t*_i_ + *N*_10_ × 100 ns).
(3-4) Reset the number of the count of the 10-MHz pulses (*N*_10_).

**Figure 1:**
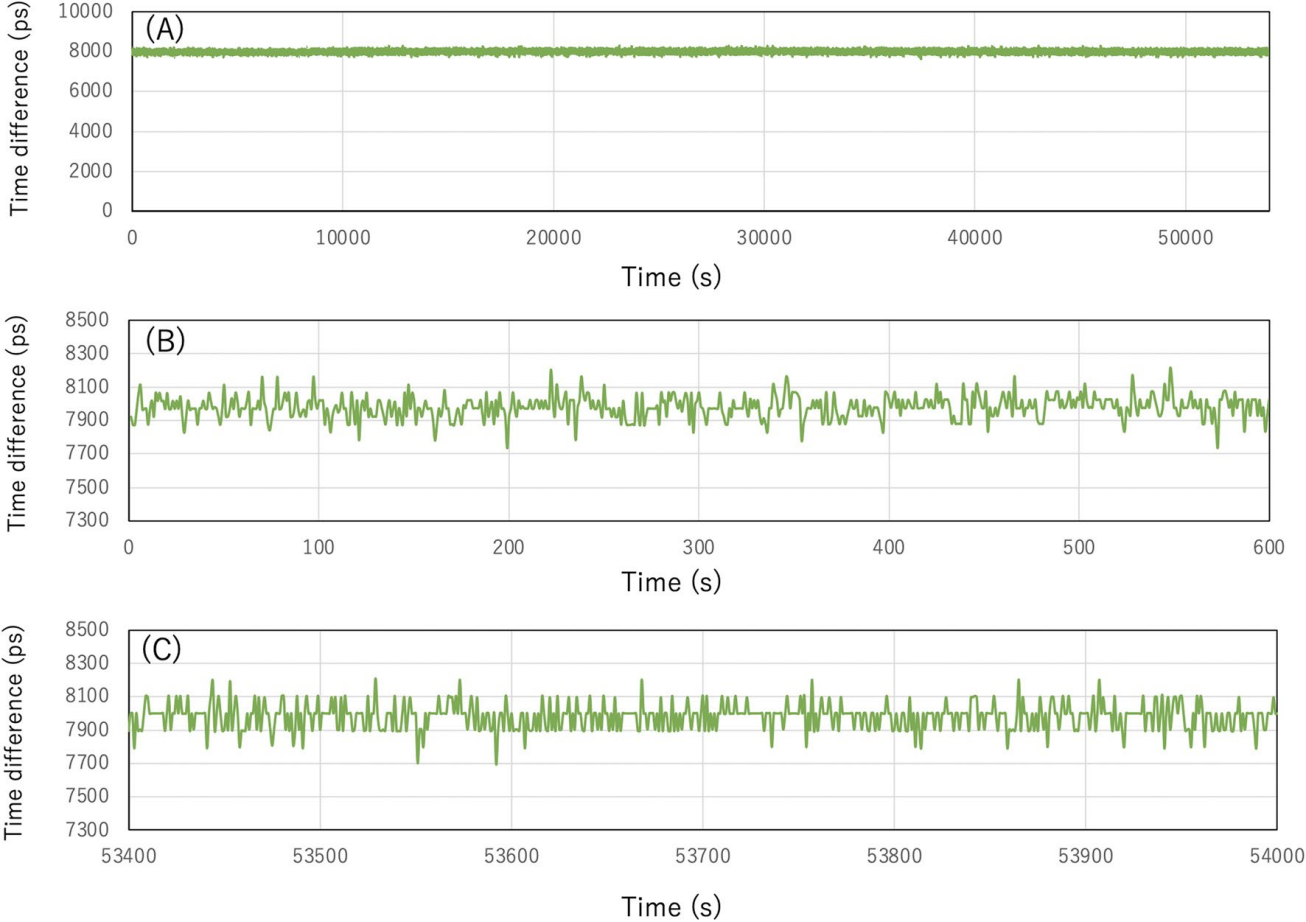
Time difference between the serial 1-Hz pulses and the 1-PPS/10-MHz pulses. The entire time series (**A**), the first 10-min time series (**B**), and the last 10-min time series (**C**) are shown.

The processes (3-3) and (3-4) are done in parallel. While a few hundred milliseconds are required to complete the (3-3) process at the maximum, the (3-4) process can also be run independently from the (3-3) process and concurrently with the (3-2) process (the GPS time labeling). The local clock drift is totally negligible within the time range of a few hundred milliseconds. As is shown in the process (3-1), since the 1-PPS/10-MHz pulses are labeled by the NMEA data, long-term stability and high accuracy of synchronization between the 1-Hz serial NMEA output and 1-PPS/10-MHz output is essential for accurately disseminating GPS time. Figure 1 shows the time difference between the serial 1-Hz pulse edge and the 1-PPS/10-MHz pulse edge outputted from the current GPS-DO (Protempis Thunderbolt™ PTP GM200). The data were collected for 15 h. In this figure, the entire time series (Fig. 1A), the first 10-min time series (Fig. 1B) and the last 10-min time series (Fig. 1C) are shown. As can be seen in this figure, these outputs are well synchronized with a granularity of 66.2 ps (S.D.) and an offset of 7.996 ns (average). No drift was observed. For the current purpose of disseminating GPS time, this quality is sufficient.

**Figure 2:**
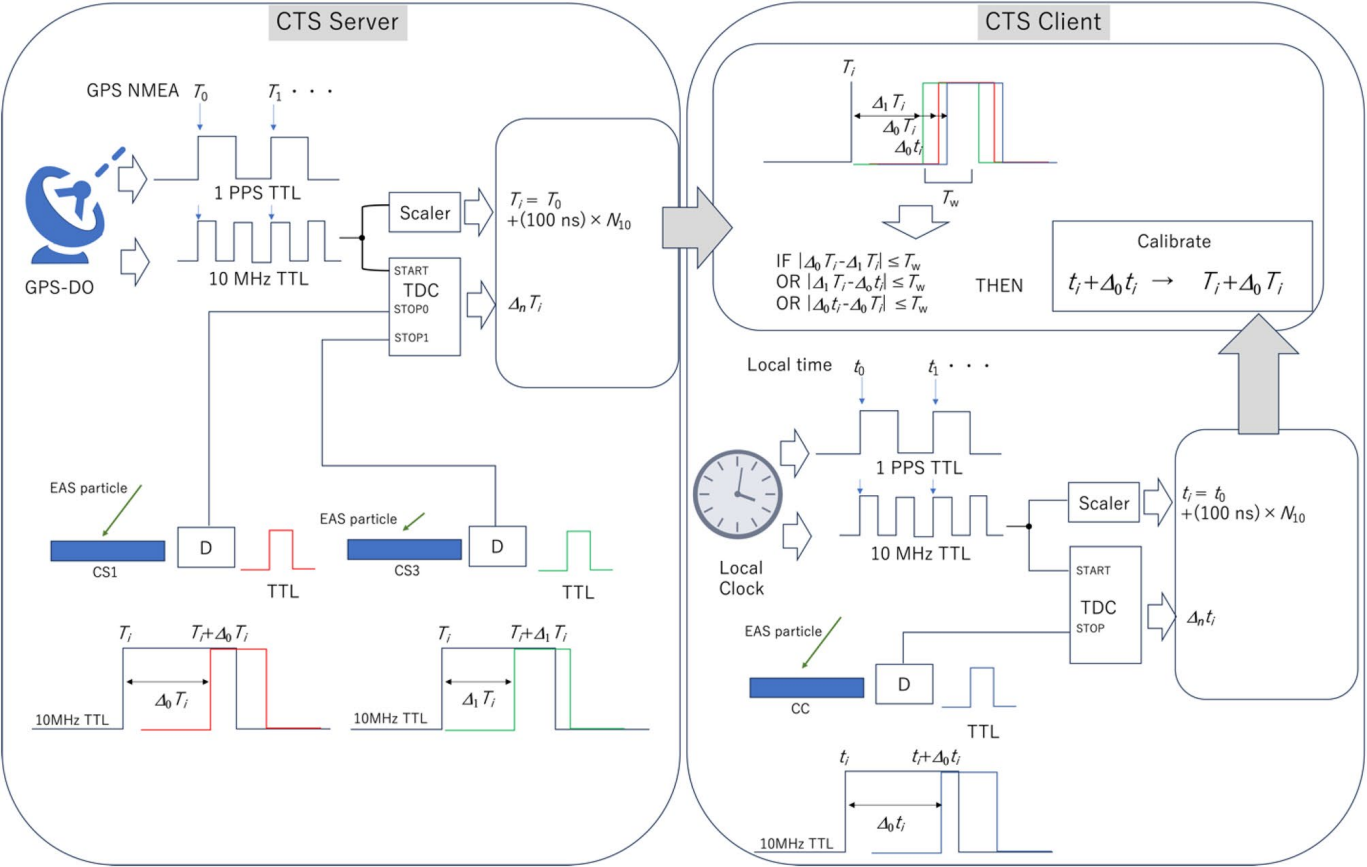
Schematic diagram of the current CTS clock correction procedure. GPS-DO, GPS-NMEA, TDC, EAS, and TTL respectively indicate the following: the GPS disciplined oscillator, the National-Marine-Electronics-Association formatted time data outputted from the GPS-DO, time to digital converter, the extended air shower, and the pulses outputted in the transistor–transistor logic format. Boxes labeled with D indicate discriminators. *T*_*i*_ and *t*_*i*_ are respectively the timestamps generated by the GPS-DO and the local clock. Other notations are described in the main text.

Many of the events detected at the CS and the CC are not particles from the targeted EAS. As a matter of fact, the majority of the particles which are detected are independent open-sky muons. Therefore, during the course of the process (2-5), accidental coincidence events between random open sky muons are sometimes generated. The capacity of the TDC we used for the current experiment is limited to a signal income rate of a few hundred Hz including background events. So, the discrimination level was adjusted so that the discrimanator’s output will be within 130–150 Hz. In the prior studies on open-sky muon measurements^27^, it was found that this discrimination level degrades the detector efficiency for charged particle detection by a factor of ~ 2 (~ 50% muons +  ~ 50% background). But here, if we assume all of these events come from random noise, we can estimate the upper limit of the accidental coincidence rate. For 150-Hz random background noise, the double-coincidence accidental rate is calculated to be 4 × 2.25 × 10^4^ × 10^–6^ = 9 × 10^–2^ Hz for the current setup (and for *T*_w_ = 2 microseconds). This means that every 25 s, an accidental coincidence can trigger an incorrect time to be distributed to the CC. Since this accidental rate is enough to impair the practical and widespread implementation of CTS, one additional backup CS is incorporated into the CTS system to reduce this error. This will be described in more detail in the Discussion section where it is shown that the same strategy also helps to improve the security of the CTS system. As an example of the effectiveness of this strategy, if one redundant CS is added to the system, the accidental rate is reduced to 12 × 3.375 × 10^6^ × 10^–12^ = 4.05 × 10^–5^ Hz; therefore, errors associated with accidental coincidences will be drastically reduced. Figure 2 shows the schematic diagram of the current CTS clock correction procedure.

### Initial CTS time synchronization procedure without GPS

In general, at the beginning of the procedure, the GPS-DO and the local clock are not synchronized very precisely (> 1 s), but we can synchronize them at a moderate level (~ 0.1 ms) by using Wi-Fi (a synchronization accuracy of 0.1 ms is achievable with Wi-Fi CERTIFIED TimeSync) and then synchronize them at a more precise level by using EAS particle events since this synchronization level (0.1 ms) is much lower than the average time interval of open-sky muon arrival (in the current case, ~ 10 ms). The clock correction procedure will be summarized as follows:

(2-1) The data from the CS is sent to the CC.
(2-2) A search for a coincidence event within a time window of 0.1 ms is done. This time window helps to reduce the number of candidates (coincidence events) to find EAS particles. The triple accidental rate will be 3 × 10^2^ × 10^2^ × 10^2^ × 10^–4^ × 10^–4^ = 3 × 10^–2^ Hz which is sufficiently lower than the open-sky muon rate.
(2-3) If a coincidence event is found, the time is transferred from the CS to the CC.
(2-4) The next data are sent from the CS to the CC.
(2-5) A search for a coincidence event within a time window (*T*_w_) of 2 microseconds is done. (In the current work, the number of signals that deviates more than 1 microsecond was studied to conform to the MiFID II requirements)
(2-6) If a coincidence event is found, the time is transferred from the CS to the CC, and this time window setting (*T*_w_ = 2 microseconds) is maintained.
(2-7) If a coincidence event is not found, the previous event is discarded, and again, a search for a coincidence event within a time window of 0.1 ms is undertaken.
(2-8) After that, repeat (2-3)–(2-7).

By repeating (2-3)–(2-7), the GPS-DO and the local clock will eventually become synchronized. For example, for the measured triple EAS coincidence rate with a detector distance of 180 m, which was 3 × 10^–3^ Hz (see the following discussions), 90% of the triple coincidence events would be accidental. If these coincidence events take place accidentally, since the clocks would not be synchronized, no coincidence events are recorded for 1/(4.05 × 10^–5^) seconds (≈ 7 h) after process (2-5). However, in practical terms, if no triple coincidence events are observed in 10 min for the process (2-7), the time window will be set back to 0.1 ms. Since the triple accidental rate is 10 times higher than the triple EAS coincidence rate for the detector distance of 180 m, if we repeat this process for 10 times, the EAS events are captured (since one out of 10 triple coincidence events comes from EAS) and the clocks are synchronized. The average time required for the initial time synchronization is 2 h (10 min × 10 times on average). Once the triple EAS coincidence events are recorded, the clock is synchronized at a sub-microsecond precision level, the following EAS events will be recorded without further corrections, and the clock will be calibrated by these following EAS events. In this work, Wi-Fi CERTIFIED TimeSync was not used. 2 GPS-DOs were used for the initial synchronization of the local clocks.

### Geometrical configuration of the experimental setup

**Figure 3:**
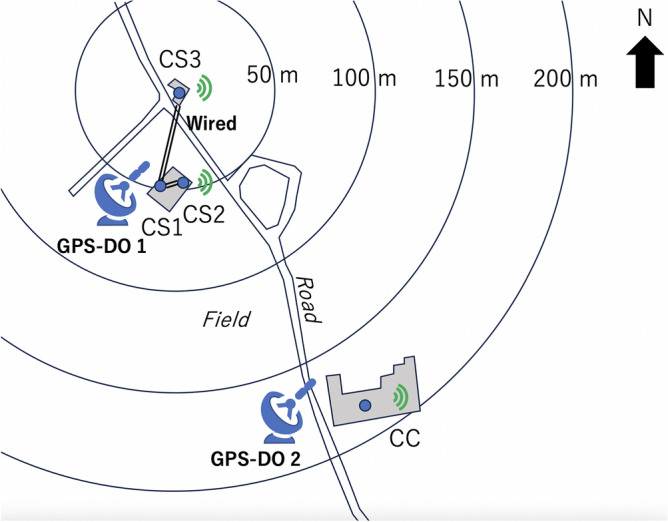
Geometrical configuration of the current experimental setup. GPS-DO1 was used for the dissemination of the reference clock, and GPS-DO2 was used for the confirmation of the accuracy. Green marks indicate the internet connection. Double lines indicate wiring between CTS stations.

**Figure 4:**
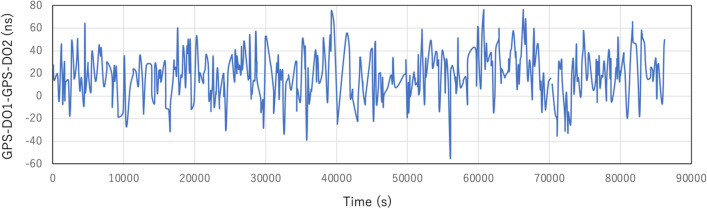
Time series of the time difference between GPS-DO1 and GPS-DO2. The measurement was performed for 24 h.

In the current work, three CTS stations were setup in Chiba Prefecture, Japan: 3 CS stations (CS1, CS2, and CS3) and one CC station (CC). In this work, the wired stations are all labeled as CTS servers. Figure 3 shows the geometrical configuration of the current experimental setup. These 3 CS stations are connected to GPS-DO1 for disseminating the GPS-DO1 time to the CC station. The distance between the CS1 and the CS3 was 50 m, and the distance between the CS3 and the CC was 180 m. All of the buildings which accommodated these CTS stations are independent of each other (not physically connected to each other). As is shown in Fig. 3, there is only a road between the CS1 and the CS2, but there is a farmer’s field between the CS1 and the CC as well as CS2 and CC. Therefore, while the CS1 and the CS2 could be wired (and thus, the results of CTS between CS1 and CS3 could be directly compared with GPS-DO1 time for the CTS accuracy evaluation), wiring a connection between the CS and the CC was not practical. In order to confirm the quality of the time synchronization between the CS1, CS3 and the CC, the CC was equipped with GPS-DO2 so that the GPS-DO1 time disseminated by CTS to the CC could be compared with GPS-DO1 time since it was assumed that current GPS-DO’s time granularity (~ 20 ns) is better than long-baseline (180 m) CTS time granularity. Figure 4 shows GPS time granularity measured with the currently adopted GPS-DO. These granularity curves were measured prior to the current demonstration by wiring two GPS-DOs: GPS-DO1 and GPS-DO2. The curves shown in this figure represent the time evolution of the GPS-DO1 with respect to GPS-DO2. As can be seen in Fig. 4, the offset and the granularity of GPS time were respectively 19.4 ns and 21.3 ns (S.D.) and that was much less than the expected accuracy of long-baseline CTS time. In this work, the triple coincidence between the CS1-CS2-CS3 and that between CS1-CS3-CC were studied. The buildings installed with the CTS system were constructed with light gauge steel (LGS). A demonstration of the time synchronization quality with the current apparatus was necessary to get closer to realizing practical implementation for real-life applications of this technique. In the current work, it was assumed that hardware was completely identical, and time delays in the transmission is the same for both. The local clock’s drift level was almost the same since the same OCXOs are used for CS and CC but as is described later, the offset was not the same for both CS and CC since the GPS-DOs we used in this work were not calibrated.

### Short/Long haul CTS measurement results

**Figure 5:**
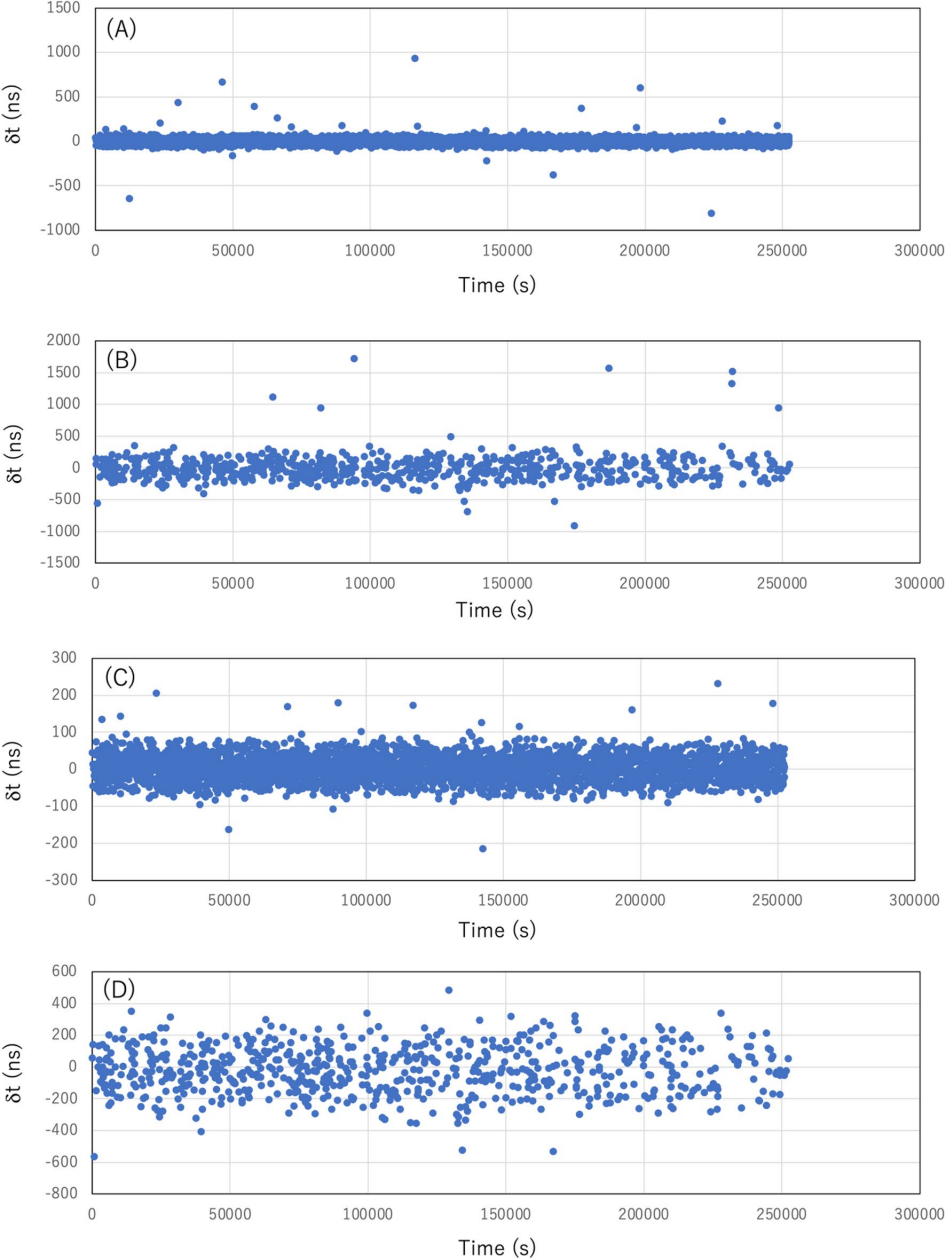
CTS measurement results. The time differences from GPS-DO1 time (*δt*) are shown for the triplet coincidence events between the CS1-CS2-CS3 (**A**) and those between the CS1-CS3-CC (**B**). The results after removing the events that deviate more than 4σ (statistically 6.3 out of 10^5^ events) from the average of the first 100 events are also shown for the triplet coincidence events between the CS1-CS2-CS3 (**C**) and those between the CS1-CS3-CC (**D**).

Figure 5 shows the current CTS measurement results. The short haul (CS1-CS2-CS3) (Fig. 5A) and long haul (CS1-CS3-CC) (Fig. 5B) results are shown. These measurements were performed for 2.52 × 10^5^ s (~ 3 days). The averaged time intervals (< *T* >) of the triplet coincidence events between the short-haul measurement and the long-haul measurement were respectively 68.1 s and 360.5 s. The drift level of OCXO in these time intervals (< 10 ns)^21^ is much smaller than the expected granularity of the CTS time. Also, the standard deviations of the time difference from GPS-DO1 (*δt*) were investigated, and these were respectively 52.1 ns for the triplet coincidence events between the CS1-CS2-CS3 and 203.4 ns for those between the CS1-CS3-CC. The number of the events that hold *δt* > 1 microsecond were respectively 0 for the triplet coincidence events between the CS1-CS2-CS3 and 5 for those between the CS1-CS3-CC. It was assumed that the events that deviate more than 4σ (statistically 6.3 out of 10^5^ events) from the average of the first 100 events were caused by the accidental coincidence events. These events were removed from each time series. As a result, the events that hold *δt* > 1 microsecond disappeared from the data between the CS1-CS3-CC. The results are shown in Fig. 5C and D. The derived accidental rates were respectively 3.9 × 10^–5^ Hz for the triplet coincidence events between the CS1-CS2-CS3, and 3.6 × 10^–5^ Hz for those between the CS1-CS3-CC, being roughly consistent with the value estimated in the previous subsection. After removing these accidental events, the average time intervals respectively became 68.3 s for the triplet coincidence events between the CS1-CS2-CS3, and 365.2 s for those between the CS1-CS3-CC. Accordingly, the standard deviations of *δt* were respectively reduced to 34.9 ns for the triplet coincidence events between the CS1-CS2-CS3 and 148.8 ns for those between the CS1-CS3-CC. This short haul result is consistent with the value reported in the previous study (35.0 ns)^18^. After the removal of the accidental events, the offset times from GPS-DO1 were respectively 3.56 ns for the triplet coincidence events between the CS1-CS2-CS3 and 22.97 ns for those between the CS1-CS3-CC. The latter offset value is consistent with the offset (19.4 ns) between GPS-DO1 and GPS-DO2 measured prior to this demonstration. These results are summarized in Table 1. In this demonstration it was shown that the precision (offset) for the long-baseline (180 m) CTS was ~ 20 ns, and the granularity was ~ 150 ns which both fit within the requirements of MiFID II the accidental rate is reduced to 12 × 10^6^ × 10^–12^ = 1.2 × 10^–5^ Hz; therefore, errors associated with accidental coincidences will be drastically reduced.

**Table 1:** Summary of short/long baseline CTS measurement results.

	Short (50 m)	Long (180 m)
Accidental rates (Hz)	3.9 × 10^–5^	3.6 × 10^–5^
< *T* > without correction (s)	68.1	360.5
< *T* > after correction (s)	68.3	365.2
*δt* without correction (ns)	52.1	203.4
*δt* after correction (ns)	34.9	148.8
Offset after correction (ns)	3.56	22.97

The measured triple coincidence rate for a distance of 50 m between the detectors were 5.3 times higher than the rate observed for a distance of 180 m. These results are roughly in agreement with the MC-based EAS simulation results (2 × 10^–1^ Hz @50 m → 2 × 10^–2^ Hz @180 m). However, the absolute values of the measured coincidence rate (1.5 × 10^–2^ Hz @50 m and 2.7 × 10^–3^ Hz @180 m) are one order of magnitude lower than the simulated value. These discrepancies were explained by the detector efficiency. Due to the limited capacity of the TDC we used in this study, the detector efficiency was significantly reduced to ~ 50%^27^; hence ~ 25% after dual coincidence and ~ 12% after triple coincidence. Further improvements in the CTS signal update rate will be expected by upgrading our TDC capacity.

## Discussion

### Applications of CTS to clock synchronization in financial districts

**Figure 6:**
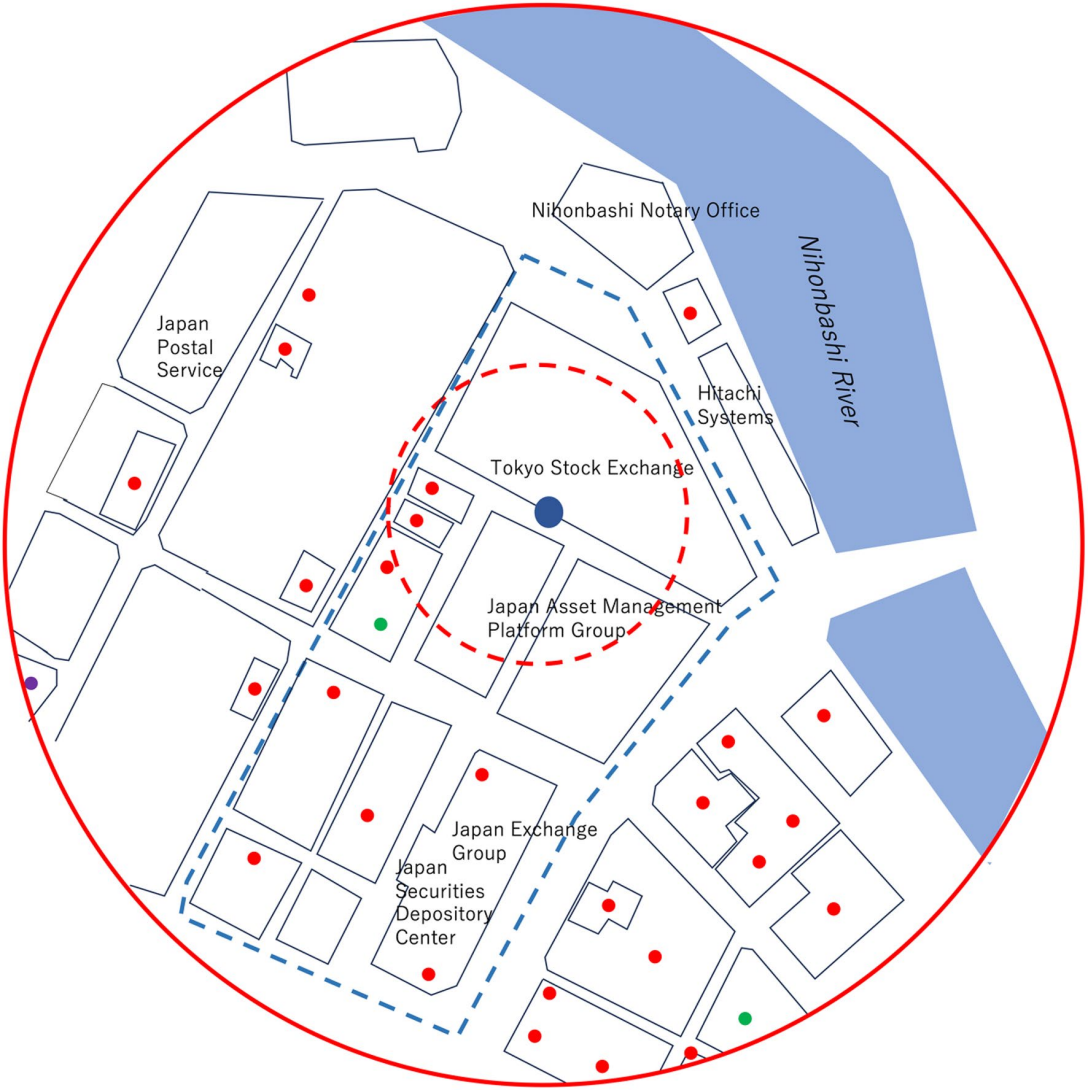
CTS area coverage in Kabutocho, Tokyo. The dashed and solid red circles respectively indicate the area within a 50 m radius and the area within a 180 m radius of the Tokyo Stock Exchange. The northern direction is oriented at the top of this map. The blue filled circle, red filled circles, green filled circles, and purple filled circles respectively indicate the positions of the CS, securities firms, banks, and insurance companies.

Securities firms (brokerage firms) handle the buying, selling and underwriting of securities such as stocks, bonds, etc. and thus, clock synchronization to UTC is essential to these firms. However, GPS connections in the Financial District are usually extremely unstable due to the fact that they are typically located in densely populated skyscrapers. Nihonbashi Kabuto-cho, Tokyo (hereafter referred to as Kabuto-cho) is the largest financial district in Japan. The Kabuto-cho area is in a typical metropolitan area^22^ where the daytime population is 8847 (mainly professional stock traders) but the night time population is drastically reduced to 168. The area includes the Tokyo Stock Exchange, Tokai Tokyo Securities, Nihon Securities, Daiwa Securities Group, Yamawa Securities, Okasan Securities Group, Yamani Securities, Phillipe Securities Japan, Kyowa Securities, Jujiya Securities, Ando Securities, and other securities firms. Stock exchanges worldwide are required to ensure highly-secured and accurate UTC synchronization; therefore, using the Tokyo Stock Exchange (TSE) and its surroundings as an example, the effectiveness of using CTS to disseminate UTC will be assessed. In this scenario, CS is installed at TSE to broadcast the CTS timestamp data (similar to a radio station broadcast) by using Wi-Fi. The clients existing at other sites in the financial district receive this Wi-Fi data and implement the processes (1-2)–(1-6) introduced in the previous section. Figure 6 shows the Kabuto-cho area, including the Tokyo Stock Exchange, indicated with a circle 360 m (solid red circle) in diameter; this is the size of the area which CTS is capable of covering, as demonstrated previously in the current experiment. For reference, a circle with a diameter of 100 m (dashed red circle) shows the area that CTS covered in its first iteration (demonstrated in the previous POC paper). The area covered by this 360 m circle includes the Tokyo Stock Exchange, 2 major banks, 1 major post office, and 27 major securities firms and other financial sector firms. As can be seen in this figure, the Kabuto-cho district (the area surrounded by the blue dashed lines) can be fully covered by this improved iteration of CTS whereas only one or two buildings could be covered with the previous iteration. As a result, 15% of all securities firms in Tokyo (182 firms)^23^ and 10% of all securities firms in Japan (268 firms) could be covered by one CTS system to effectively transfer time within the guidelines of UTC.

**Figure 7:**
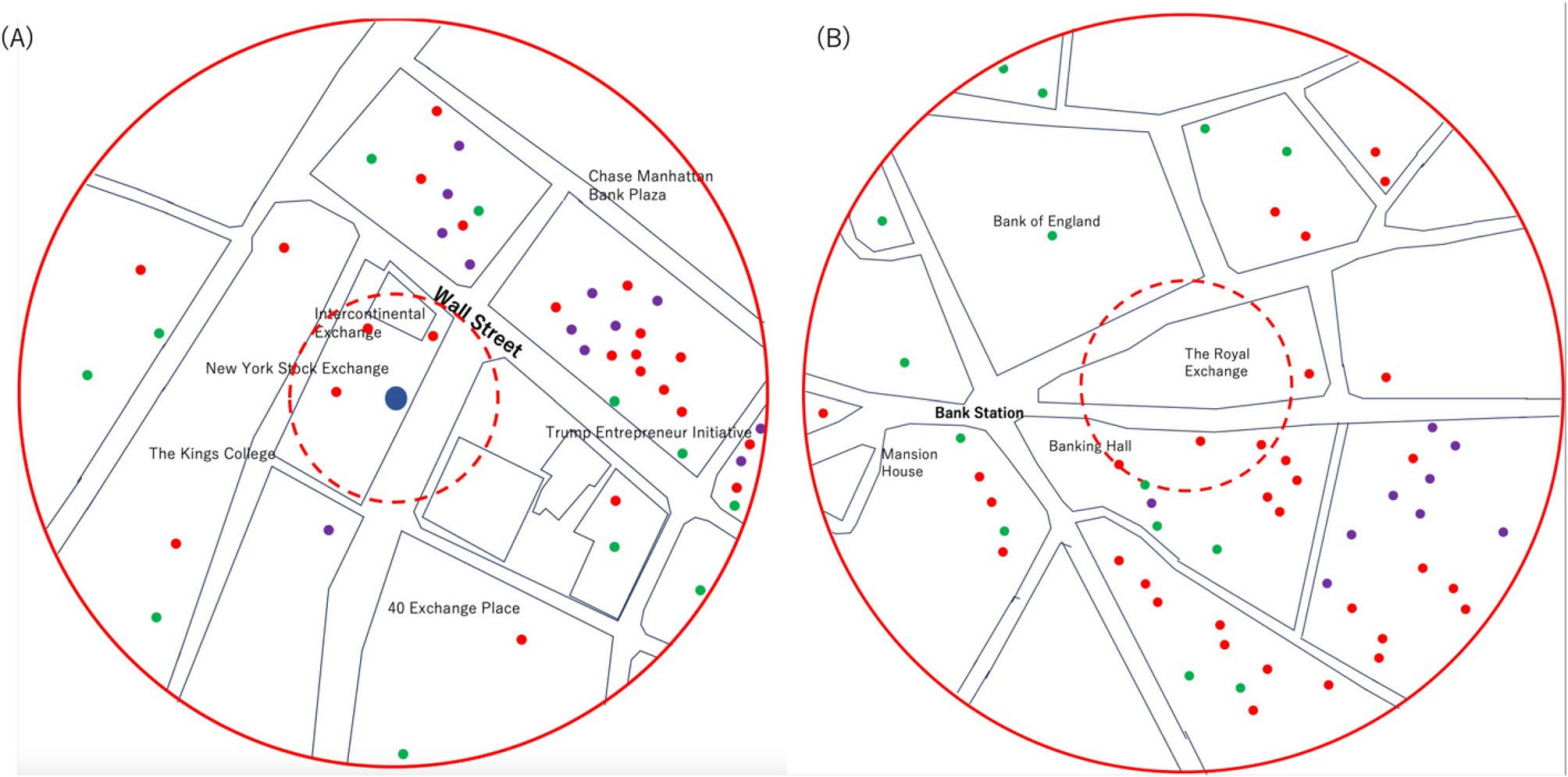
CTS area coverage in Wall Street, New York (**A**) and Lombard Street, London (**B**). The dashed and solid red circles respectively indicate the area within a 50 m radius and the area within a 180 m radius of the New York Stock Exchange and the Royal Exchange. The northern direction is oriented at the top of this map. The blue filled circle, red filled circles, green filled circles, and purple filled circles respectively indicate the positions of the CS, securities firms, banks, and insurance companies.

Likewise, estimations of one CTS system’s coverage area in Wall Street, New York and Lombard Street, London were also investigated. Wall Street, New York is the largest financial district in the USA. The area includes a number of financial sectors including the New York Stock Exchange (NYSE), Intercontinental Exchange, Citi Bank, and Chase Bank. Lombard Street, London is the largest financial district in the UK and includes the Royal Exchange (RE), the Bank of England, NatWest Group, and British Arab Commercial Bank, along with other banks and financial centers. As can be seen in Fig. 7A, a large number of securities firms are located within an area 2 blocks north of NYSE. The distance from NYSE to this cluster of securities firms ranges from 50 to 180 m, and the concentration of the securities firms are drastically reduced outside this area. On the other hand, as can be seen in Fig. 7B, (compared to the NYSE) there is a higher concentration of the number of securities firms in a few blocks south of the RE. The distance from the RE to this cluster of the securities firms also ranges from 50 to 180 m. Similar to New York, only a few security firms exist in the blocks outside of this area. As a result, it was confirmed that the current CTS system with its expanded range could cover most of the financial clusters associated with the stock exchanges in New York, London, and Tokyo.

### CTS security

For applications of CTS to clock synchronization in financial districts, one of the major concerns would be malicious attempts to crack into CS broadcasting data to modify the time information and to distribute incorrect time information to the clients. The general trend of our modern society is directed towards increasing the use of wireless networks. Current wireless networks are more flexible and convenient than wired networks, but they also tend to have more security risks. Wireless links are more vulnerable to malicious third-party attackers; thus, wireless security is becoming more and more important to strengthen in order to protect our modern networks and data^24^. For the networks using IEEE-1588 Precision Time Protocol (PTP), research has already demonstrated that clock synchronization data can be modified by malicious third-party attackers using a combination of an Address Resolution Protocol (ARP) “poisoning attack” followed by a “delay attack”^25^. Similar attacks could be directed towards CTS. However, even in this case, incorrect time information cannot be delivered to the clients using CTS. CTS is robust against such an attack for the following reasons. Although the CTS timestamp data broadcasted from the CS can be peaked and rewritten by a third party in principle (a so-called man-in-the-middle attack), the third party will never know which timestamp is the timestamp registered by the particles in the same EAS. A majority of the events broadcast by CS are random open-sky muon events that cannot be used for CTS. Therefore, a third-party attacker would need to change all of these timestamps to send the wrong timing information to the CC. Moreover, even if this third party was to add 100 microseconds to all of the timestamps, these timestamps would not be verified with the timestamps at the CC since it wouldn’t coincide with the events recorded at the client locations. Consequently, the data at the CC can only be verified by the data broadcasted from the authorized CS. However, a third-party attacker could also attempt to highjack the CS. If the CS stopped broadcasting the CTS data, the local clock associated with the CC would not be able to properly calibrate its clock, so eventually the clock would drift. This problem can be mitigated by adding redundant backup CSs at the stock exchanges since it would be exponentially more difficult to highjack multiple CSs at the same time.

As a case study, the third party attempts to generate fake timestamps to interfere with the CTS system. Here we assume malicious party broadcasts fake CTS signals to generate accidental coincidence events with EAS events within a microsecond time window. There is a possibility that this malicious party will generate fake events that accidentally coincide with EAS events by broadcasting artificially generated random signals. However, this action is unpractical for the following reason. Since 1-MHz random events will respectively coincide with every EAS event, the malicious party needs to broadcast CTS signals at a rate of 1-MHz (a rate which is much higher than the open-sky muon rate) and thus, these fake signals can be easily distinguished from the true CTS signals. If the malicious party thins out the broadcasting rate to 100 Hz, which is comparable to the open-sky muon rate, these random events will coincide with 1 EAS event for every 10,000 EAS events. However, such coincidence events are not practically useful for delivering fake timestamps for the following reason. Since the clock's drift is continuous, if the delivered CTS timestamps strongly deviate from the expected time deviation (from the clock’s drift level), these CTS timestamps are treated as erroneous timestamps and are rejected. Therefore, in order to deliver fake timestamps which will not be discarded, the malicious party needs to provide these timestamps within this deviation range. On the other hand, the malicious party also needs to provide timestamps that are largely deviated from UTC to damage the CTS users. In order to do so, the malicious party has to continuously deliver these fake timestamps so that they gradually deviate from the true timestamps. As a result of this action two timelines (UTC and fake time) are generated at the CTS users. However, even if the malicious party could achieve this step, since there won’t be a high enough fake coincidence rate with the EAS events (one out of every 10,000 EAS events), the fake timeline can be easily distinguished from the UTC timeline.

### CTS costs

The costs which would be required to install and maintain CTS are also one of the major concerns for judging the feasibility of utilization for practical applications. Securities firms already install stable time servers such as the GPS-disciplined Rubidium-based time servers. These time servers can trace GPS time and can holdover its time when GPS signals are temporally lost. Their holdover quality is ~ 1 microsecond for 24-h operation without the GPS signals. The costs of these time servers are 20–30k dollars. On the other hand, each CC unit costs 8k dollars. Its breakdown is as follows: (A) a plastic scintillator sheet: 2k dollars, (B) a PMT: 1k dollars, (C) the TDC module: 4k dollars, and (D) associated electronics such as the HV module, the discriminator, OCXO, etc.: 1k dollars. When considering CTS offers totally wireless and GPS-free clock synchronization service, this cost would be sufficiently competitive in the clock synchronization market. Like GPS, CTS receives signals from the sky, but CTS receives signals originating from cosmic rays whereas GPS receives signals originating from Cs clocks which are inside satellites. CTS doesn’t require any active source and thus, the electric power consumption is small. The main electronic components of CTS are PMT and TDC; these are parts that are relatively robust and are designed for long-term operation. It is anticipated this new CTS system can be easily adapted to real-world applications since detectors with a similar configuration have been working for many months without any maintenance^26^, therefore, it is anticipated that the CTS maintenance costs are minimal.

In conclusion, it was confirmed that the current achievements in the quality of CTS (an offset of 22.97 ns (including the GPS-DO’s offset) and a granularity of 148.8 ns) fulfills the MiFID II requirements (an offset of < 100 microseconds and a granularity of < 1 microsecond) for high-frequency trades, and those in the CTS range (180 m) can cover a majority of the clusters of financial sectors in New York, London, and Tokyo. The next step is to continue operating this system in this newly constructed facility for longer durations (years) to confirm the long-term stability of this system and prepare CTS for implementation to diverse applications worldwide.

## Data Availability

The datasets used and/or analyzed during the current study available from the corresponding author on reasonable request.
